# Calcium and potassium channels are involved in curcumin relaxant effect on tracheal smooth muscles

**DOI:** 10.1080/13880209.2020.1723647

**Published:** 2020-03-25

**Authors:** Bahman Emami, Farzaneh Shakeri, Zahra Gholamnezhad, Saeideh Saadat, Marzie Boskabady, Vahab Azmounfar, Hamed Sadatfaraji, Mohammad Hossein Boskabady

**Affiliations:** a Neurogenic Inflammation Research Center, Mashhad University of Medical Sciences, Mashhad, Iran; b Natural Products and Medicinal Plants Research Center, North Khorasan University of Medical Sciences, Bojnurd, Iran; c Department of Physiology, School of Medicine, Mashhad University of Medical Sciences, Mashhad, Iran; d Department of Physiology, School of Medicine, Zahedan University of Medical Sciences, Zahedan, Iran; e Dental Materials Research Center and Department of Pediatric Dentistry, School of Dentistry, Mashhad University of Medical Sciences, Mashhad, Iran; f Department of Pharmacognosy, School of Pharmacy, Mashhad University of Medical Sciences, Mashhad, Iran

**Keywords:** Relaxation, isometric contraction, bronchodilator agents, ion channels

## Abstract

**Context:**

Curcumin, the active component of *Curcuma longa* L. (Zingiberaceae), exhibits a wide variety of biological activities including vasodilation and anti-inflammation.

**Objective:**

The relaxant effect of curcumin in tracheal smooth muscle (TSM) was not examined so far, thus, this study was designed to assess the relaxant effect of curcumin on rat TSM and examine the underlying mechanism(s) responsible for this effect.

**Materials and methods:**

TSM was contracted by KCl (60 mM) or methacholine (10 μM), and cumulative concentrations of curcumin (12.5, 25, 50, and 100 mg/mL) or theophylline (0.2, 0.4, 0.6, and 0.8 mM, as positive control) were added to organ bath. The relaxant effect of curcumin was examined in non-incubated or incubated tissues with atropine (1 μM), chlorpheniramine (1 μM), indomethacin (1 μM), and papaverine (100 μM).

**Results:**

In non-incubated TSM, curcumin showed significant relaxant effects on KCl-induced contraction in a concentration-dependent manner (*p* < 0.001 for all concentrations). The relaxant effects of curcumin 12.5, 25, and 50 mg/mL were significantly lower in atropine-incubated tissue compared to non-incubated TSM (*p* < 0.05 to *p* < 0.001). A significant difference was observed in EC_50_ between atropine-incubated (48.10 ± 2.55) and non-incubated (41.65 ± 1.81) tissues (*p* < 0.05). Theophylline showed a significant relaxant effect on both KCl and methacholine-induced contraction in a concentration-dependent manner (*p* < 0.001 for all cases).

**Conclusions:**

The results indicated a relatively potent relaxant effect of curcumin on TSM, which was less marked than the effect of theophylline. Calcium channel blocking and/or potassium channel opening properties of curcumin may be responsible for TSM relaxation.

## Introduction

Curcumin, the main yellow pigment of *Curcuma longa* L. (Zingiberaceae), exerts a wide variety of biological activities including anti-inflammatory (Menon and Sudheer 2007), antioxidant (Vajragupta et al. 2004; Hassani et al. 2015), anticarcinogenic (Kakarala et al. 2010)/anticancer (Mirzaei et al. 2016), antidepressant (Huang et al. 2011), hepatoprotective (Park et al. 2000), antidiabatic (Yang et al. 2015), antibacterial (Di Mario et al. 2007), antifungal (Khalil et al. 2012), memory enhancing (Tabrizian et al. 2019), nephroprotective (Rezaee et al. 2017), and antiulcer (Tuorkey and Karolin 2009) activities. Other biological activities of curcumin including suppression of the key enzymes such as phospholipase, lipoxygenase, cyclooxygenase 2, collagenase, elastase, and hyaluronidase, and inhibition of leukotrienes, thromboxane, prostaglandins, nitric oxide, monocyte chemoattractant protein-1, interferon-inducible protein, tumour necrosis factor alpha (TNFα) and interleukin-12 (Chainani-Wu 2003) were demonstrated in various *in vitro* and *in vivo* studies. These effects may contribute to curcumin anti-inflammatory properties (Camacho-Barquero et al. 2007; Claramunt et al. 2009).

The respiratory effects of curcumin were investigated in several *in vitro* and *in vivo* studies (Pillai et al. 2004; Lüer et al. 2011). It was indicated that curcumin decreased apoptosis in human lung cancer cell lines in a concentration-dependent manner (Pillai et al. 2004). Curcumin showed antibacterial activity in upper respiratory tract epithelial cells, through reducing bacterial growth, inhibiting bacterial adherence and invasion, and inhibiting pro-inflammatory cytokine release from upper respiratory tract epithelial cells (Lüer et al. 2011).

Curcumin also showed a concentration-dependent relaxant effect on isolated porcine coronary arteries (Xu et al. 2007). This effect was mediated through NO, cGMP, and β-adrenergic receptors (Xu et al. 2007). In addition, pre-treatment with curcumin showed significant vasodilatory effects in diabetic rat aorta (Nurullahoğlu-Atalık et al. 2012).

The relaxant effect of hydro-ethanol extract of *C. longa* on rat tracheal smooth muscle and its possible mechanisms was shown in a previous study (Emami et al. 2017). Results showed that the relaxant effect of the extract was not due to its effect on β-adrenergic, muscarinic, or histamine (H1) receptors, calcium or potassium channels, NO production, or phosphodiesterase activity, but this effect is perhaps due to xanthine like property or a non-adrenergic, non-cholinergic effect. *C. longa* contains curcumin, demethoxycurcumin, and bisdemethoxycurcumin, as well as volatile oils (i.e., tumerone, atlantone, and zingiberone), sugars, proteins, and resins (Emami et al. 2017). However, curcumin is the main constituent of *C. longa*, and the relaxant effect of the plant on tracheal smooth muscle could be due to this agent (Emami et al. 2017). The relaxant effect of curcumine in tracheal smooth muscle was not examined so far, thus, the current study was designed to assess the relaxant effect of curcumin on rat tracheal smooth muscle (TSM) and examine the underlying mechanism(s) responsible for this effect.

## Materials and methods

### Chemicals

Methacholine, atropine, chlorpheniramine, indomethacin, and papaverine were purchased from Sigma Chemical Ltd, UK. Potassium chloride and other substances for preparing Krebs-Henseleit solution were obtained from Merk, Germany.

### Animals

Fifty-five young male Wistar rats (200–250 g) were purchased from the Animal House, School of Medicine, Mashhad University of Medical Sciences (Mashhad, Iran). The animals were maintained under controlled conditions with 12 h light/dark cycle at 22 ± 2 °C. Water and food were always accessible *ad libitum* to animals. The study was designed according to the regulations set by the ethics committee of Mashhad University of Medical Sciences and approved by the committee (Approval No. 930658).

The present study was performed on male rats because there may be gender-dependent differences in trachea smooth muscle contraction or relaxation. Oestrogen was reported to produce relaxation in smooth muscles of gall bladder, urinary bladder, blood vessels, stomach, colon and trachea (Al-Shboul et al. 2019).

### Tissue preparation

For obtaining the trachea, the rats were sacrificed by a blow on the neck and the chest was opened and excess of connective tissue and fat were dissected out (Holroyde 1986). A piece of the trachea with 5–6 cartilage rings was isolated and mounted between two stainless steel hooks in 10 mL organ bath containing Krebs-Henseleit solution, and maintained at 37 ± 0.5 °C with isometric tension of 1 g as previously described (Saadat, Yasavoli, et al. 2019). The isometric transducer (MLT0202, AD Instruments, Australia) linked to a power lab system (Power Lab 8/30, ML870, AD Instruments, Australia) was used to measure the contraction and relaxation responses in all experiments (Emami et al. 2017).

### Protocol

Cumulative concentrations of curcumin (12.5, 25, 50, and 100 mg/mL), (Sami Labs Ltd., Bangalore, India) or theophylline (0.2, 0.4, 0.6, and 0.8 mM, used as a positive control) were added to pre-contracted TSM at 5 min intervals, to produce concentration-response curves. As a negative control, the effect of 1 mL saline (NS) on pre-contracted TSM was also evaluated in each experiment. The effective concentration of curcumin causing 50% of maximum response (EC_50_) was obtained from the concentration-response curve in each experiment using 50% of the maximum response in the Y axis and the concentration of curcumin causing this response in the X axis (Saadat, Naghdi, et al. 2019). Cumulative concentrations of curcumin were chosen based on the previous studies (Emami et al. 2017).

### Experimental groups

To examine the relaxant effect of curcumin, non-incubated, or incubated tissues with different substances for 10 min, were contracted with KCl or methacholine for 5 min (Emami et al. 2017). The relaxant effect of curcumin was evaluated in various groups as described in Table 1.

**Table 1.** The protocol of the study and the methods of evaluating of various mechanisms of the relaxant of effect of curcumin on TSM.

Contracture agent	Condition	Incubating substance	Mechanisms
60 mM KCl	Non-incubated tissues (*n* = 10)		
	Incubated tissues	1 μM atropine (*n* = 11)	Muscarinic receptor inhibition
		1 μM chlorpheniramine (*n* = 8)	Histamine (H_1_) receptor inhibition
		1 μM indomethacin (*n* = 8)	Cyclooxygenase inhibition
		100 μM papaverine (*n* = 8)	Phosphodiesterase inhibition
10 μM methacholine	Non-incubated tissues (*n* = 10)		

The relaxant effect of curcumin in each group was examined in 8–11 tracheal rings *in vitro*, each trachea from different animals. The effect of theophylline as positive control, was only examined in non-incubated tissues.

### Data analysis

Data are presented as mean ± SEM. The results were analysed using one-way analysis of variance (ANOVA) followed by Tukey’s Multiple Comparison test. Statistical significance was set at *p* < 0.05.

## Results

### The relaxant effect of curcumin in KCl-contracted TSM

**Figure 1.**
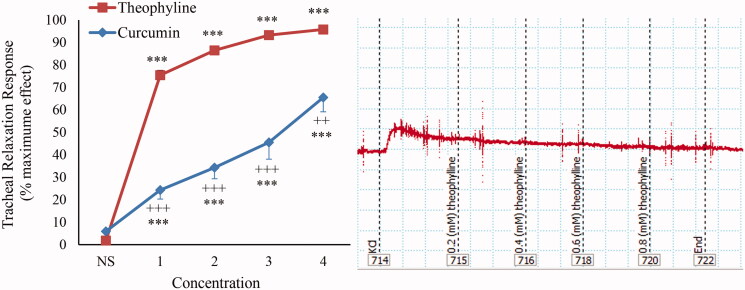
Concentration-response relaxant effect (mean ± SEM) of curcumin (*n* = 10) and theophylline (*n* = 9) on KCl (60 mM) induced contraction of TSM in non-incubated tissues and its trace sample. ***p < 0.001 compared to saline (NS); +++*p* < 0.001 compared to the effect of theophylline.

In non-incubated TSM contracted by KCl (60 mM), a significant relaxant effect was seen for curcumin in a concentration-dependent manner (*p* < 0.001 for all concentrations) (Figure 1).

**Figure 2.**
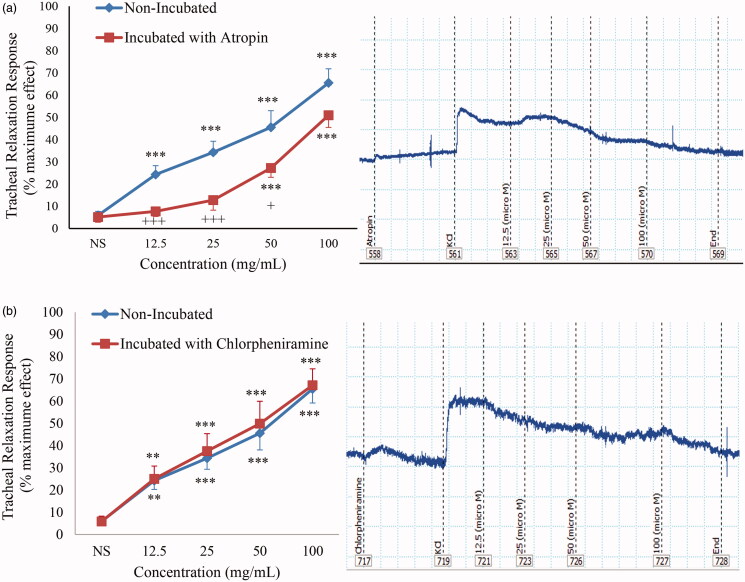
Concentration-response relaxant effect (mean ± SEM) of curcumin on KCl (60 mM) induced contraction of TSM in non-incubated (*n* = 10) and incubated tissues with atropine (1 μM, *n* = 11) (a) and chlorpheniramine (1 μM, *n* = 8) (b), and their trace samples. **p < 0.01; ***p < 0.001 compared to saline (NS); +*p* < 0.05; +++*p* < 0.001, compared to non-incubated tissues.

Curcumin at concentrations of 50 and 100 mg/mL showed significant and concentration-dependent relaxant effects in atropine (1 μM)-incubated TSM (*p* < 0.001) (Figure 2(a)). The relaxant effects of curcumin 12.5, 25, and 50 mg/mL were significantly lower in atropine-incubated tissue compared to non-incubated TSM (*p* < 0.05 to *p* < 0.001).

In addition, curcumin showed significant and concentration-dependent relaxant effects in chlorpheniramine (1 μM)-incubated TSM (*p* < 0.001 for all concentrations), (Figure 2(b)). There were no significant differences in the relaxant effects of curcumin between non-incubated and chlorpheniramine-incubated tissues.

**Figure 3.**
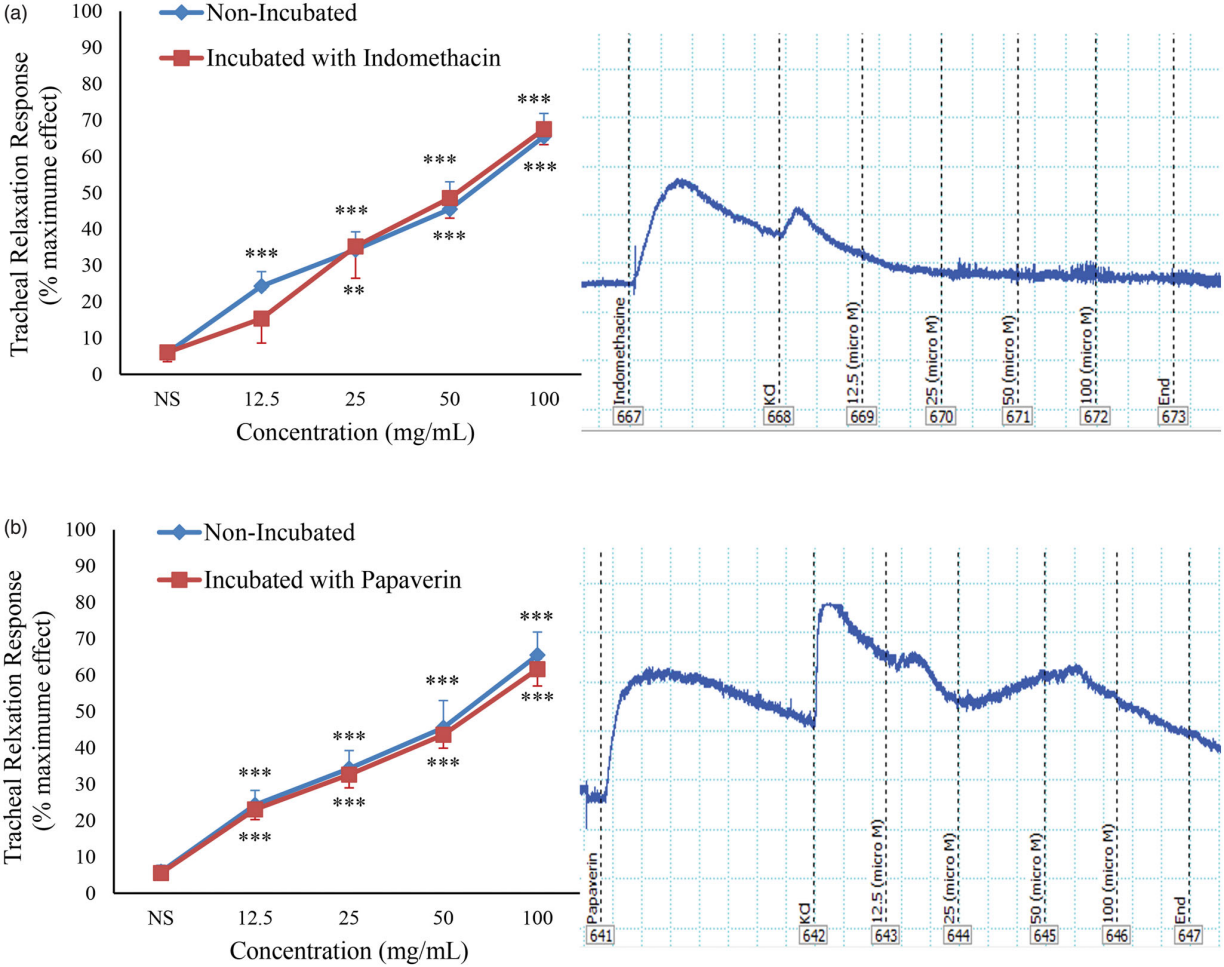
Concentration-response relaxant effect (mean ± SEM) of curcumin on KCl (60 mM) induced contraction of TSM in non-incubated (*n* = 10) and incubated tissues with indomethacin (1 μM, *n* = 8) (a) and papaverin (100 μM, *n* = 8) (b), and their trace samples. **p < 0.01; ***p < 0.001 compared to saline (NS).

In indomethacin (1 μM)-incubated tissues, a significant and concentration-dependent relaxant effect was seen for curcumin (*p* < 0.001), (Figure 3(a)). No significant difference was found in the relaxant effects of curcumin between non-incubated and indomethacin-incubated tissues.

Moreover, a significant and concentration-dependent relaxant effect was observed for curcumin in papaverine (100 μM)-incubated TSM (*p* < 0.001), (Figure 3(b)). The relaxant effect of different concentrations of curcumin did not show statistically significant difference between non-incubated and papaverine-incubated tissues.

**Figure 4.**
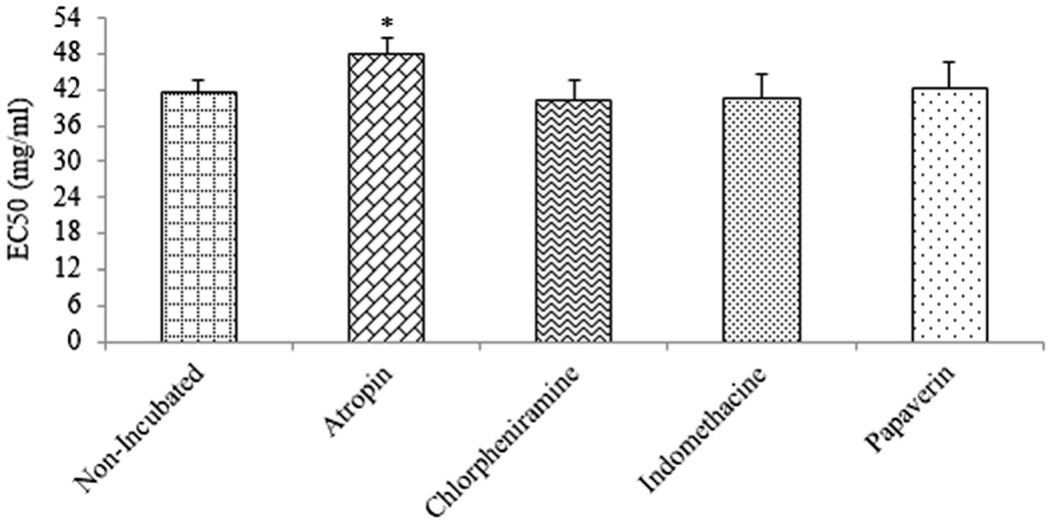
EC_50_ values of curcumin induce relaxation obtained from contracted TSMs of rat with 60 mM KCl in non-incubated (*n* = 10) and incubated tissues with atropine (*n* = 11), chlorpheniramine (*n* = 8), indomethacin (*n* = 8) and papaverine (*n* = 8). There was not significant difference in EC_50_ values between non-incubated and incubated tissues atropine.

No significant difference was found in curcumin EC_50_ values between non-incubated tissues (41.65 ± 1.81) and tissues incubated with chlorpheniramine (40.12 ± 3.62), indomethacin (40.74 ± 3.96) and papaverine (42.36 ± 4.25). A significant difference was only observed for EC_50_ in atropine-incubated tissue (48.10 ± 2.55) compared to non-incubated tissues (Figure 4).

There was also no statistically significant difference in the relaxant effects of various concentrations of curcumin, among TSM incubated with different substances (atropine, chlorpheniramine, indomethacin and papaverine) in KCl-induced muscle contraction (Table 2).

**Table 2.** Comparison of the relaxant effect curcumin (percentage change in proportion to the maximum contraction) in different incubated TSM contracted by 60 mM KCl, (*n* = 9).

Incubating substance	Concentration (mg/mL)			
	12.5	25	50	100
Atropine	42.74 ± 4.46	61.78 ± 7.18	82.10 ± 5.98	86.57 ± 7.03
Chlorpheniramine	49.60 ± 5.92	75.46 ± 4.90	91.31 ± 2.89	98.47 ± 0.75
Indomethacin	36.72 ± 3.09	60.28 ± 2.41	85.65 ± 3.07	96.99 ± 2.13
Papaverine	48.63 ± 5.02	66.60 ± 3.82	79.48 ± 3.83	91.25 ± 2.12

Data were presented as mean ± SEM. There was no any significant difference in the relaxant effect of curcumin between various incubated tissues.

### The relaxant effect of curcumin in methacholine-contracted TSM

**Figure 5.**
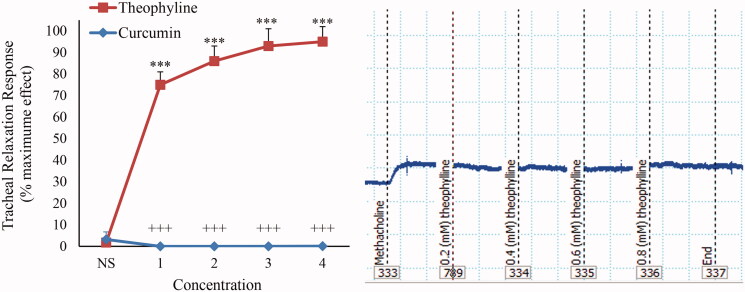
Concentration-response relaxant effect (mean ± SEM) of curcumin (*n* = 10) and theophylline (*n* = 9) on methacholine (10 μM) induced contraction of TSM in non-incubated tissues and its trace sample. ***p < 0.001 compared to saline (NS); +++*p* < 0.001 compared to the effect of theophylline.

In non-incubated TSM contracted by methacholine (10 μM), curcumin did not induce any relaxant effect (Figure 5).

**Figure 6.**
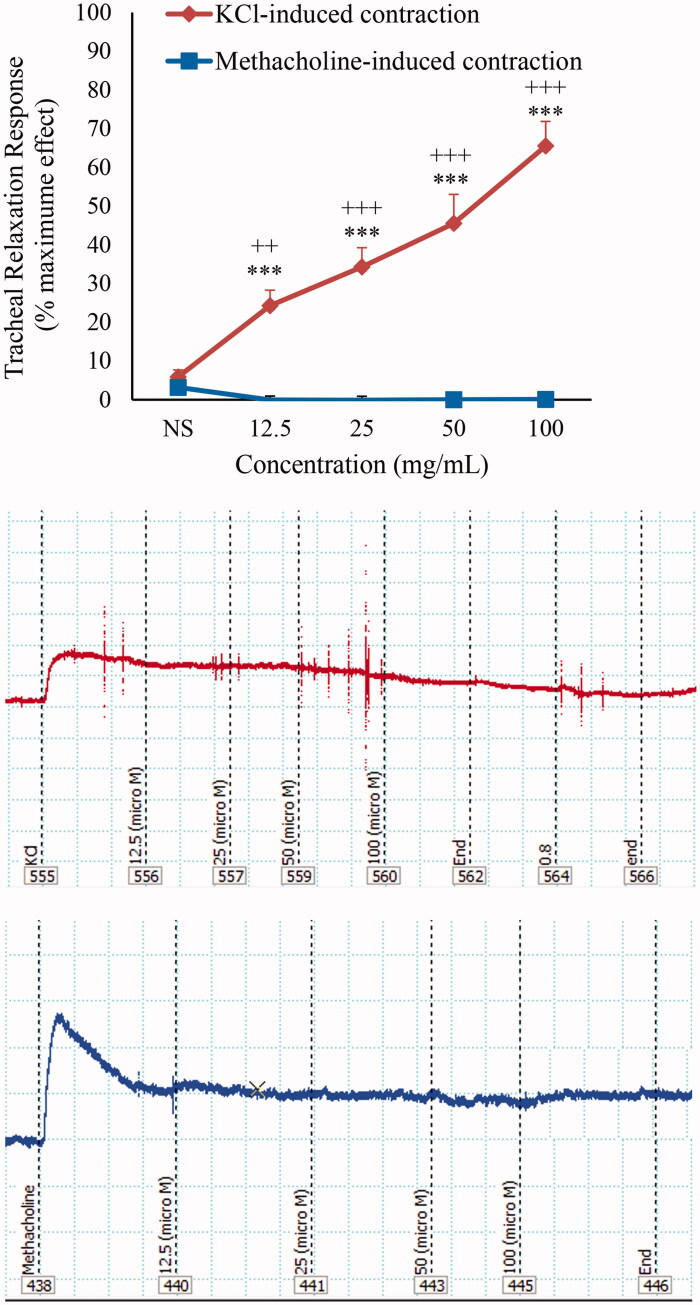
Concentration-response relaxant effect (mean ± SEM) of curcumin on KCl (60 mM) and methacholine (10 μM) induced contraction of non-incubated TSM (*n* = 10) and their trace samples, respectively. ***p < 0.001 compared to saline (NS); +++*p* < 0.001; compared to the relaxant effect in methacholine induced muscle contraction.

The relaxant effects of various concentrations of curcumin on KCl-contracted TSM were significantly higher than those of methacholine-induced contraction (*p* < 0.01 to *p* < 0.001) (Figure 6).

### Comparison of the relaxant effects of curcumin and theophylline

The relaxant effects of all concentrations of curcumin were significantly lower than that of theophylline in KCl-induced contraction of non-incubated TSM (*p* < 0.01 to *p* < 0.001) (Figure 1). The relaxant effects of all concentrations of theophylline were significantly higher than those of curcumin in methacholine-contracted TSM (*p* < 0.001 for all concentrations) (Figure 5).

### Correlations between the relaxant effect of curcumin and theophylline and their concentrations

Significant correlations were seen between the relaxant effects of theophylline (*r* = 0.823, *p* < 0.001) and curcumin (*r* = 0.652, *p* < 0.001) and their concentrations in non-incubated tissues contracted by KCl (Table 3). There was significant correlations between the relaxant effect of curcumin and its concentrations in KCl-induced contraction of TSM incubated with atropine (*r* = 0.776, *p* < 0.001), chlorpheniramine (*r* = 0.584, *p* < 0.001), indomethacin (*r* = 0.623, *p* < 0.01) and papaverine (*r* = 0.757, *p* < 0.001) (Table 3).

**Table 3.** Relationship between the relaxant effect of curcumin and theophylline with their concentrations in different experimental groups.

Contractile agents	Studied agents	Conditions	R	*p* Value
KCl	Curcumin	Non-Inc	0.652	*p* < 0.001
		Atropine-Inc	0.776	*p* < 0.001
		Cholerphenirmine-Inc	0.584	*p* < 0.001
		Indomethacin-Inc	0.623	*p* < 0.001
		Papverine-Inc	0.757	*p* < 0.001
	Theophylline	Non-Inc	0.823	*p* < 0.001
Methacholine	Curcumin	Non-Inc	0.323	*p* > 0.05
	Theophylline	Non-Inc	0.615	*p* < 0.001

Data were presented as mean ± SEM.

## Discussion

The relaxant effects of curcumin in rat TSM might be mediated by different mechanisms, including stimulation of β_2_-adrenergic receptors (Linden et al. 1993), inhibition of histamine H_1_ receptors (Popa et al. 1984), calcium channel-blocking effects (Miyahara et al. 1993), potassium channel-opening effects (Buckle et al. 1993), inhibitory effects on muscarinic receptors (Loenders et al. 1992), methylxanthine activity (Boskabady et al. 2004), NOS inhibition (Danser et al. 2000), phosphodiesterase inhibition (Shimizu et al. 2000), cyclooxygenase inhibition (COX) (Slattery et al. 2001), and ATP-sensitive potassium channels inhibition (Satoh et al. 1991). To evaluate some of these possible mechanisms, the relaxant effect of curcumin was examined in TSM incubated with atropine, chlorpheniramine, papaverine, and indomethacin.

The relaxant effect of muscarinic antagonists in TSM was previously documented (Loenders et al. 1992). Therefore, in atropine-incubated TSM contracted by KCl, the contribution of the muscarinic receptor inhibition in the curcumin relaxant effect was evaluated. The findings showed significantly lower relaxant effects for 12.5, 25, and 50 mg/mL of curcumin in tissues incubated with atropine compared to non-incubated TSM. These results indicated the inhibitory effect of curcumin on muscarinic receptors, which might contribute to its relaxant effect in TSM.

To examine the role of histamine (H_1_) receptors and the effect of curcumin on these receptors, the relaxant effect of curcumin was evaluated on chlorpheniramine-incubated TSM contracted by KCl. The relaxant effect of histamine (H_1_) antagonists in TSM was previously shown (Popa et al. 1984). The findings of this group showed that the relaxant effect of curcumin did not vary significantly between incubated and non-incubated TSM. These results indicated that curcumin is not a histamine (H_1_) antagonist and lacks the inhibitory effects on histamine (H_1_) receptors.

In papaverine-incubated TSM, the relaxant effect of curcumin was studied to examine the role of phosphodiesterase inhibitory mechanism. In previous studies, the relaxant effect of phosphodiesterase inhibiting agents on TSM was demonstrated (Shimizu et al. 2000). No significant difference was seen in the relaxant effects of curcumin between non-incubated and papaverine-incubated tissues. Therefore, curcumin is not a phosphodiesterase inhibitor and phosphodiesterase inhibition does not contribute to the relaxant effect of curcumin in TSM.

To evaluate possible involvement of prostacyclin pathway in curcumin-induced TSM relaxation, the effect of curcumin was also examined in indomethacin-incubated tissues. The anti-inflammatory effect of curcumin (Jacob et al. 2007) might be mediated through its cyclooxygenase inhibitory effect. Non-significant differences in the relaxant effect of curcumin between non-incubated and indomethacin-incubated tissues might show that prostacyclin pathway is not involve in the curcumin induced TSM relaxation.

Higher EC_50_ values of curcumin induced relaxant effects and lower relaxant effects of curcumin obtained in the atropine-incubated tissues compared to non-incubated tissues also support the contribution of muscarinic receptor inhibitory property of curcumin to TSM relaxation effect. However, curcumin did not show any significant relaxant effect on methacholine-induced contraction of TSM. The absence of the relaxant effects of curcumin in methacholine-induced contraction of smooth muscle almost excluded a muscarinic antagonism effect for curcumin-induced tracheal smooth muscle relaxation. The discrepancy between the effects of curcumin on methacholine-induced TSM contraction and KCl-induced muscle contraction in atropine-incubated tissues is unclear and should be evaluated in further studies.

The absence of relaxant effects of curcumin on methacholine-induced muscle contraction and relatively potent relaxant effects on KCl-induced TSM contraction may indicate that the relaxant effect of curcumin is mainly mediated via its inhibitory effect on calcium channels and/or opening effect on potassium channels. Previously, the relaxant effects of potassium channel opening drugs (Buckle et al. 1993; Laurent et al. 1993; Miura et al. 1993) and calcium channel blocking drugs (Sonna et al. 1996) on TSM were shown. Significant correlations between the relaxant effects of curcumin and its concentrations also support its relatively potent and concentration-dependent relaxant effect in TSM.

Previous studies showed the relaxant effect of curcumin in various types of smooth muscle, which support the findings of the present study (Itthipanichpong et al. 2003; El-Sayed 2008; Cheng et al. 2010). Curcumin significantly inhibited isolated guinea-pig ileum and rat uterus pre-contracted with acetylcholine, histamine, and KCl through receptor-dependent and independent mechanisms (Itthipanichpong et al. 2003). The concentration-dependent relaxant effects of curcumin were previously investigated in isolated caprine urinary bladder detrusor muscle contracted by acetylcholine. In another study, curcumin enhanced contractility in urinary bladder isolated from rats via a stimulatory effect on muscarinic receptors (Cheng et al. 2010). The relaxant effect of curcumin was also indicated in rat uterus (El-Sayed 2008). The possible mechanisms responsible for curcumin relaxant effects, were examined by tetraethyl ammonium, a potassium channel blocker, glibenclamide, an ATP-sensitive potassium channel blocker, and propranolol, a β-adrenergic receptor blocker. Findings showed that the relaxant effect of curcumin is mediated by stimulatory effects on β_2_-adrenergic receptors, inhibitory effects on muscarinic receptors, and the opening of ATP-sensitive potassium channels. The discrepancy regarding possible mechanisms underlying the relaxant effect of curcumin on smooth muscle observed between El-Sayed and our study, may be due to the types of smooth muscle (caprine detrusor muscle vs. TSM) and differences in the distribution of various receptors and channels in these two different types of muscle.

In conclusion, the present study showed a relatively potent relaxant effect for curcumin in TSM which was lower compared to the effect of theophylline at studied concentration. The findings also suggested that calcium channels blocking and/or potassium channels opening effects of curcumin may be responsible for its relaxant effect in TSM.
